# Sharing Science: Enabling Global Access to the Scientific Literature

**DOI:** 10.1289/ehp.119-a520

**Published:** 2011-12-01

**Authors:** Adrian Burton

**Affiliations:** Adrian Burton is a biologist living in Spain who also writes regularly for *The Lancet Oncology*, *The Lancet Neurology*, and *Frontiers in Ecology and the Environment*.


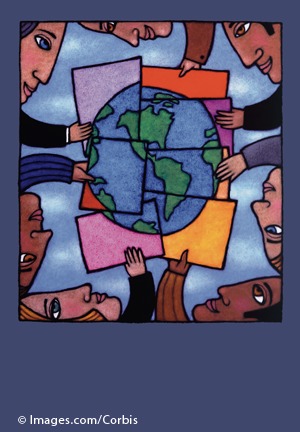
Peer-reviewed literature is the formal channel of communication for the scientific community. Through it, scientists convey their discoveries to one another across distance and time. Providing both a broadcast system and an archive, it is pivotal to the collaborative effort that is modern science. Without access to it, a scientist cannot keep up with develop-ments, has nowhere to contribute findings, and is pretty much out of the loop.

One traditional problem of access to the literature faced by researchers and academics in developing countries has been the inability of their institutions to afford journal subscriptions, which can run into the thousands of dollars per publication. But things have been changing, with the last 10 years having seen important efforts to make the world’s peer-reviewed scientific journals available to these members of the research community either free or at a much-reduced price. Top-flight open-access journals have also come into being, theoretically making the papers they contain free for all to use. But is purchase price the only obstacle hindering the entry of the developing world’s researchers into international scientific dialogue?

## The Need for Access

Summer 2011 saw the tenth anniversary of six major biomedical publishers (Blackwell, Elsevier Science, the Harcourt Worldwide STM Group, Wolters Kluwer International Health & Science, Springer Verlag, and John Wiley) coming together under the coordination of the World Health Organization (WHO) to announce the launch of HINARI (Health InterNetwork Access to Research Initiative; also known as HINARI Access to Research in Health Programme). HINARI’s mission was to provide free or very low-cost online access to some 1,500 biomedical and social science journals for public, nonprofit institutions in emerging nations.

The need was clear: a WHO survey undertaken in 2000 showed that 56% of institutions in countries with a gross national income per capita (GNIpc) of under US$1,000 had no current subscription to any international journal.^1^ In comments made in honor of the tenth anniversary of HINARI’s launch, WHO director general Margaret Chan said of the survey, “Researchers and academics in developing countries ranked lack of access to the top medical and scientific literature as one of their most pressing problems. The reason was straight-forward: lack of money to pay for subscriptions.”^2^

The HINARI program grew, and with 160 publishers on board it now provides some 8,000 health and biomedical information sources (mainly peer-reviewed journals) free to the institutions of 63 countries with a current GNIpc of US$1,600 or less.^3^ For 42 other countries with a GNIpc of US$1,601–4,700, access is offered for US$1,000 per year per institution.

Sister program AGORA (Access to Global Online Research in Agriculture), involving more than 40 publishers and managed by the Food and Agriculture Organization of the United Nations in partnership with Cornell University, was launched in 2003 and now provides online access to more than 1,200 food science–related journals under the same conditions. In 2006 OARE (Online Access to Research in the Environment), involving more than 150 publishers and managed by the United Nations Environment Programme in partnership with Yale University, began to provide the same type of access to over 3,000 journals covering the environmental sciences. And in 2009 the World Intellectual Property Organization (WIPO) launched its Access to Research for Development and Innovation program, which offers low-cost access to more than 200 science and technology journals from 12 publishers to eligible patent offices and research institutes.

Together, HINARI, AGORA, OARE, and WIPO, collectively known as Research4Life, are now making journals available to more than 5,000 user institutions.^4^ Similar initiatives including Stanford University’s HighWire,^5^ INASP (International Network for the Availability of Scientific Publications),^6^ and open-access ventures such as *EHP* and the Public Library of Science (PLoS) stable of publications, have also improved access to scientific information.

“Access to information is the key to unlocking the participation of scientists in low- and middle-income countries in the global scientific discourse,” explains HINARI program manager Kimberly Parker, who is based in Geneva, Switzerland. “Without the latest evidence relevant for their work and research, our colleagues in the Global South^7^ may waste precious time repeating known research or may develop policies based on outdated knowledge.”

The Research4life website holds a telling testimonial written by Shehu U. Abdulahi, vice-chancellor of Ahmadu Bello University in Zaria, Nigeria: “A few years ago we carried out an experiment for surgical operations of some livestock animals, and as we thought it was excellent research we wrote a manuscript on the findings for publication in a journal. However, after a review of the manuscript it came back with the comment that the drug which we used as anaesthesia for the animals had been banned about five years earlier. Had we had access to up-to-date published literature through such resources like AGORA, this would not have happened.”^4^

Arun Neopane, editor-in-chief of the *Journal of the Nepal Paediatric Societ*y, points to the public health benefits conferred by access to the literature. “A couple of years ago universities, libraries, medical colleges, and research organizations in Nepal did not have easy access to medical literature relating to research from most of the peer-reviewed journals,” he says. “Previously one could only access the abstracts found in PubMed/MEDLINE®. With access to HINARI the vast repository of medical literature suddenly opened up for us, and there was no looking back.” Neopane says this access has helped medical personnel in their clinical and community practices and indirectly is even helping the country achieve its Millennium Development Goals.^8^

## Working the Web

But simply making journals affordable and available online may not be enough. To view an online journal you first need a computer and an Internet connection that can cope with the data flow. “Computer availability can be a real stumbling block in some countries,” explains Sue Silver, editor-in-chief of *Frontiers in Ecology and the Environment*, who with a colleague runs workshops in China on how to get published in top Western journals. “In some institutions a computer may need to be shared between a large number of people, so access time may be limited.”

The sharp end of this problem can be severe: a 2007 report indicated that Internet cafes were the main point of connection to the web for postgraduate doctors in Lagos Teaching Hospital in Nigeria and Yaoundé University in Cameroon.^9^ “In addition, in some countries electricity supplies are unreliable, so a computer and its router may not always be working. And then there are the added problems of a fast-enough connection to make browsing possible and high connection costs,” Silver adds.

Connection speeds can indeed be a serious problem. *EHP*, which publishes a Chinese edition as part of its International Program,^10^ discovered that many Chinese readers were having problems downloading large PDF files because they only had access to dial-up modems or low-bandwidth connections. This problem was circumvented when the journal began publishing the online version of its Chinese edition in HTML.

Generally speaking, the situation is improving, with broadband Internet access becoming more widely available, more affordable, and faster. “New undersea cables are providing Africa with more international Internet bandwidth,” explains Vanessa Gray, senior information and communications technology analyst at the International Telecommunication Union (ITU) in Geneva. “With more and more countries in Africa launching 3G mobile broadband services, an increasing number of people have access to the Internet at high speed.”

But although these services help increase coverage and offer mobility, Gray says they usually provide only limited data access at lower speeds, which makes them unsuitable for intensive users such as researchers or institutions. So-called fixed broadband services, which are much faster, are usually limited to urban areas and remain very expensive, she says, explaining, “ITU’s tariff data suggest that the cost of an entry-level fixed broadband connection in Africa often exceeds the average monthly per-capita income.”^11^

Even when they have a good connection, researchers may need training in how to use online resources. “Researchers who have had limited Internet access may simply not know how to search for information or even know that PubMed [and other resources] exist,” Silver says. A 2010 report showed that researchers’ ability to utilize electronic resources at four African universities was limited by the use of unsophisticated search strategies.^12^ Further, a report published in 2007 indicated that only 47% of those surveyed at four African teaching hospitals knew HINARI existed.^9^

The solution may be training. “Research4Life focuses on information training needs by providing a range of options from running short courses to self-paced training modules as well as some distance education courses,” Parker says. “[But] in the end, it will be regional and local organizations such as ITOCA^13^ that will turn the corner for scientists to use our information resources and other Internet tools.”

## Research Output

Access to the literature, however, is not just about being able to see it; it’s also about contributing to it. At the 2009 World Conference of Science Journalists Research4Life reported that its three initiatives had spurred a dramatic rise in research output by scientists in developing countries.^14^ Analyses showed that absolute growth in research output between 1996 and 2002 was 25% in what would then have been non-Research4Life countries (i.e., countries that would have been ineligible to register given their GNIpc at that time), 22% in countries that would have been eligible for free access, and 30% in those that would have been eligible for low-price access. Between 2002 and 2008, however, with Research4Life under way, these figures increased to 67%, 145%, and 194%, respectively.

Although some authors have questioned the reliability of these numbers,^15^ regarding them as speculative due to methodolo-gical limitations, there would appear to be little doubt that these initiatives have helped researchers in the developing world see their work published. Najeeb Al-Shorbaji, director of knowledge management and sharing at WHO headquarters in Geneva, says HINARI has improved the quality of publications, increased the number of indexed articles, and enhanced research capacity in participating countries.

Unfortunately, lack of training in proper manuscript preparation remains an important hurdle to many researchers in developing countries.^16^ But help is available in this area through organizations such as AuthorAID,^17^ an INASP program that helps researchers in developing nations improve their science communication skills through personal mentoring and workshops on best practices in scientific writing and publishing. Developing-world researchers may also face a lack of interest in the areas in which they work by international journals.^16^ They must then publish in nonindexed national journals, leaving their work largely invisible. And of course, the language barrier can hinder many non-Anglophone scientists as most international journals are published in English.^18^ Even when these problems can be overcome, the obstacle of author submission fees may still remain.

The African Journal Partnership Project^19^ has been tackling many of these problems for nearly a decade by partnering selected African journals with mentor journals in the United States and Great Britain. For example, *Ghana Medical Journal* has partnered with *The Lancet*, *Malawi Medical Journal* with *JAMA*, and *Mali Médical* with *EHP*. Such partnerships have raised awareness of these journals’ existence and enhanced their quality and therefore their chances of becoming indexed.^20^

For instance, *EHP* contributes toward its partner’s visibility by hosting the online version of the Malian journal, providing training in online publishing, and sending lecturers to Mali to conduct regional workshops on manuscript preparation, peer review, and publishing. “Thanks to the partnership project, *Mali Médical* has had its own website, managed by *EHP*, since 2004, and the journal has been indexed in MEDLINE and PubMed since 2008,” says Siaka Sidibé, editor-in-chief of *Mali Médical*. “With these two steps, *Mali Médical* is more and more visible, and the journal is disseminated worldwide.”

Other initiatives, such as the Journals Online Project, overseen by INASP’s Programme for the Enhancement of Research Information,^21^ are raising the visibility of national journals in Latin America, Asia, and Africa. The WHO and its regional offices are also compiling databases in the form of Regional Index Medici and a Global Index Medicus.^22^ These web-based indices of medical and health journals published in WHO member states offer better access to and visibility for journals not included in international indices such as MEDLINE.

Ten years after the start of HINARI, access to the scientific literature would now appear to be less of a problem for researchers in developing countries—but the problem has not gone away altogether. Only with further infrastructural and economic development at the national policy level and appropriate training might it be truly removed, Al-Shorbaji says. This, and understanding that the scientists of developed nations have no monopoly on ideas and knowledge, should be encouragement enough to search for new ways to improve the engagement of our colleagues in the Global South.
